# Dynamic Change of VOR and Otolith Function in Intratympanic Gentamicin Treatment for Ménière's Disease: Case Report and Review of the Literature

**DOI:** 10.1155/2013/168391

**Published:** 2013-02-26

**Authors:** L. E. Walther, R. Huelse, K. Blättner, M. B. Bloching, A. Blödow

**Affiliations:** ^1^Department of Otorhinolaryngology, Head and Neck Surgery, University Medicine Mannheim, Ruprecht-Karls-University Heidelberg, 68135 Mannheim, Germany; ^2^Department of Otorhinolaryngology, Head and Neck Surgery, Helios Clinic Berlin-Buch, 13125 Berlin, Germany

## Abstract

Intratympanic gentamicin treatment (IGT) is an evidence-based therapeutic option for recurrent vertigo attacks in Ménière's disease (MD). Today, in MD it is possible to monitor changes of vestibular receptor function, induced by IGT, with objective test methods such as the video head impulse test (vHIT) and cervical and ocular vestibular evoked myogenic potentials (cVEMP, oVEMP) in a dynamic, time-and frequency-dependent manner. We report on a 65-year-old female patient with recurrent vertigo attacks in a right-sided MD, where receptor function was followed up before and up to 4 weeks after IGT (time dynamic). Quantitative changes of vestibular function (frequency dynamic) were detected with bithermal calorics and vHIT, with air-conducted sound (ACS) cVEMP and bone-conducted vibration (BCV) oVEMP at 500 Hz. The horizontal vestibuloocular reflex (hVOR) gain in vHIT decreased successively until the 4th week with the appearance of catch-up covert and catch-up overt refixation saccades, and side asymmetry increased in caloric testing. Saccular function was extinguished within 4 weeks, whereas utricular function was diminished after 4 weeks. Monitoring vestibular receptor function with objective test methods provides a quantitative insight into the dynamic activity of vestibular function and is therefore applicable in order to adjust IGT regimen at different therapeutic stages.

## 1. Introduction

Gentamicin plays an important role in otorhinolaryngology due to its ototoxic side effects. Intravenous administration may cause severe uni- or bilateral vestibular loss with imbalance and oscillopsia. If administered intratympanically, gentamicin vestibulotoxicity can be used to eliminate recurrent vertigo attacks in cases of intractable Ménière's disease (MD) [1–3]. For many patients, even at a higher age, intratympanic gentamicin treatment (IGT) is an evidence-based therapeutic option to restore quality of life [1, 2]. In IGT either a partial deficit or a complete loss of vestibular receptor function may occur.

Today, canal and otolith functions are capable of being measured objectively and quantitatively by means of the video head impulse test (vHIT), cervical and ocular vestibular evoked myogenic potentials (oVEMP, cVEMP) [4–7]. These new tests provide the opportunity to monitor IGT-induced changes of vestibular function. Additional methods such as subjective visual vertical (SVV) and caloric irrigation may be helpful to confirm the functional status. When these tests are performed in a follow-up setting, a dynamic insight in receptor function with regard to time periods (dynamic of time) and frequency working range of receptor function (dynamic of frequency) can be yielded.

In this article we want to highlight the diagnostic results in a patient with recurrent vertigo attacks in MD, who was successfully treated with IGT. Changes of the horizontal vestibuloocular reflex (hVOR), sacculocollic and utriculoocular reflexes were controlled dynamically by means of vHIT, bithermal irrigation, cVEMP, oVEMP, and SVV in a frequency- and time-dependent manner.

## 2. Case report

A 65-year-old female was diagnosed with MD on the right side 6 years ago. Diagnosis of definite MD was made according to the criteria of AAO-HNS since the patient complaint of tinnitus and rotational vertigo (up to several hours) and a fluctuating hearing loss were documented. For the last 8 months she suffered from several severe disabling vertigo attacks, up to at least twice per week. Medication with cinnarizine and Diamox as well as betahistine in a high-dosage regimen (3 × 48 mg) did not reduce the symptoms effectively. The quality of life assessed with dizziness handicap inventory (DHI) deteriorated within the last few months. IGT was recommended, and therefore 12 mg of gentamicin was instilled into the tympanic cavity after surface anaesthesia (4% Lidocaine) of the tympanic membrane.

**Figure 1:**
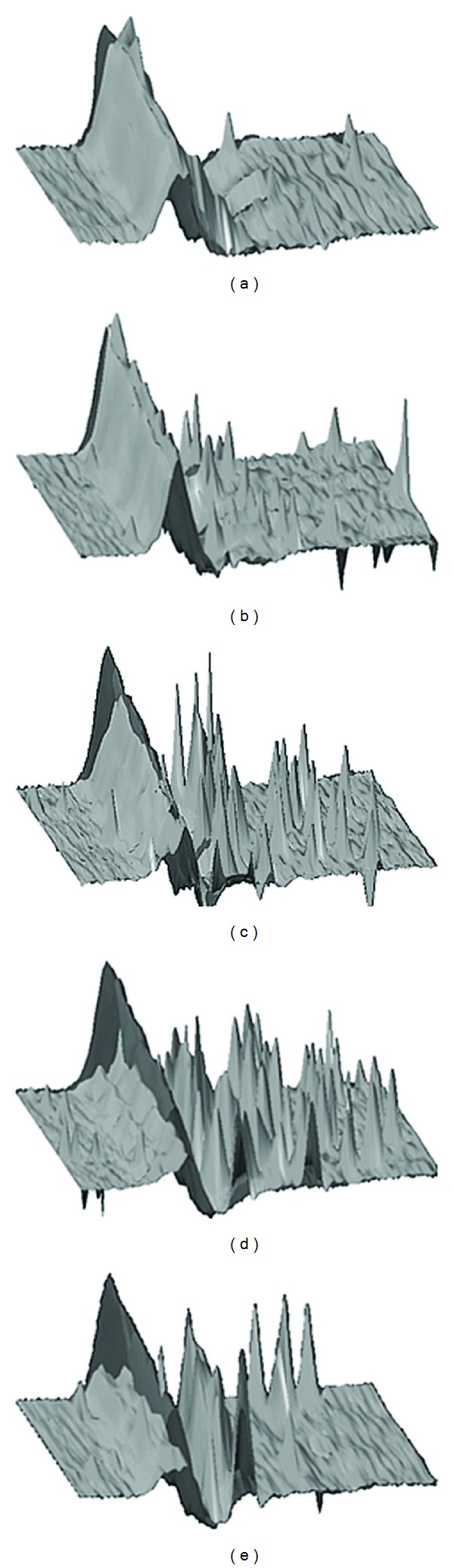
Monitoring of dynamic changes of hVOR with the vHIT in intratympanic gentamicin treatment. (a) Before therapy, (b)–(e) 1–4 weeks after therapy. Grey: hVOR: eye acceleration, black: head acceleration. Time-dependent reduction of hVOR gain and appearance of overt and covert refixation saccades.

Hearing (pure tone audiogram) and vestibular tests (bithermal caloric irrigation, vHIT, cVEMP, oVEMP, and SVV) were performed at the same time. Before gentamicin treatment, the pure tone audiogram showed a sensorineural hearing loss on the right side (mean hearing loss at 0.5, 1, 2, and 3 kHz = 63.75 dB). It changed until the 4th week after therapy (mean hearing loss = 75 dB). HVOR was measured in vestibular low-frequency range using bithermal caloric irrigation with water (44° and 20° Celsius) in supine position. Before treatment, the side difference was 46% (normal <25%) but deteriorated to 75% within 4 weeks after treatment. High-frequency changes of hVOR were monitored using the vHIT. Before IGT, the vHIT revealed a gain of 0.95 (normal hVOR gain >0.68) and few physiological saccades. One week after IGT, the hVOR gain was reduced but was normal at 0.86 and few compensational refixation saccades (catch-up covert) occurred. At week 2, the hVOR gain was deficient at 0.36 and decreased further to 0.25 and 0.21 at weeks 3 and 4 with a clear appearance of catch-up overt saccades (Figure 1).

**Figure 2:**
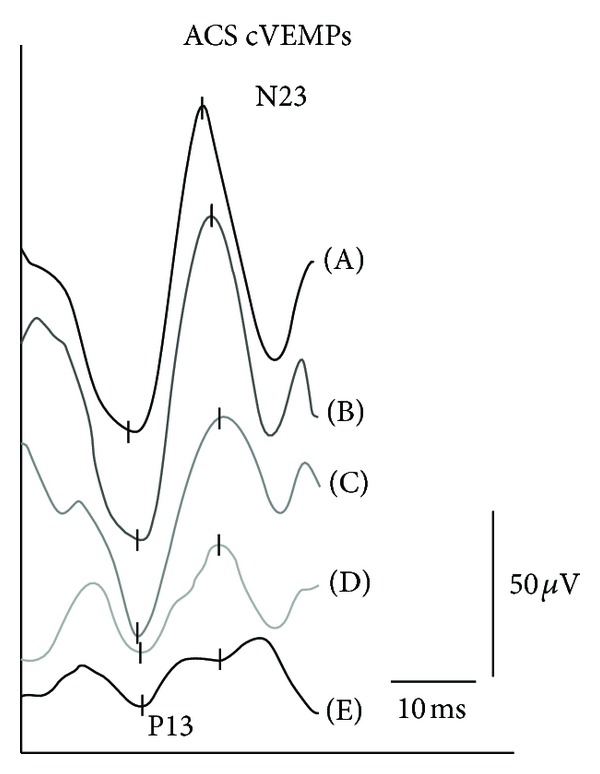
ACS cVEMP at 500 Hz (100 dB nHL) before (A) and after intratympanic gentamicin treatment from week 1 (B) to week 2 (C), week 3 (D), and week 4 (E).

**Figure 3:**
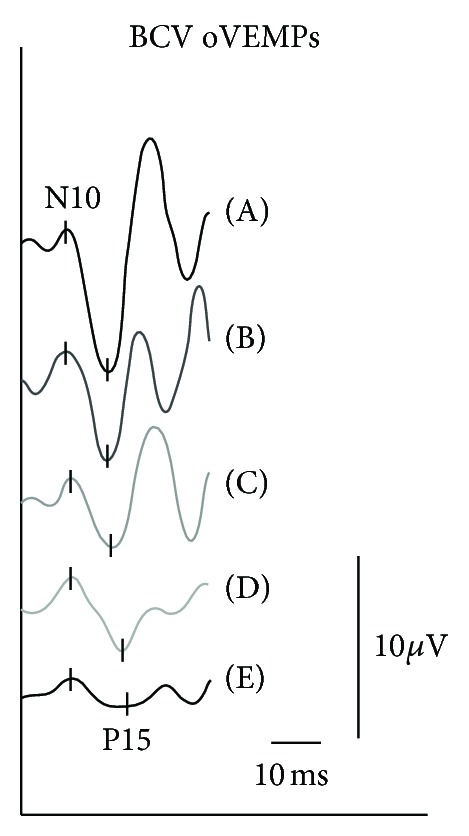
BCV oVEMP at 500 Hz (100 dB nHL) before (A) and after intratympanic gentamicin treatment from week 1 (B) to week 2 (C), week 3 (D), and week 4 (E).

**Figure 4:**
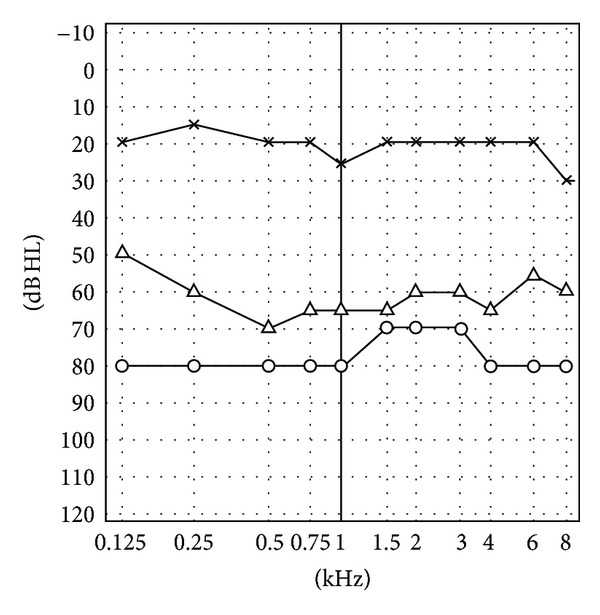
Pure tone audiogram before (open triangle) and 4 weeks after intratympanic gentamicin treatment (open circle) in right-sided Ménière's disease, unaffected left side (asterix).

Otolith function was measured with using air-conducted sound stimulation (ACS) cVEMP (500 Hz), with bone-conducted vibration (BCV) oVEMP (500 Hz) using a hand-held minishaker 4810 (Bruel&Kjaer, Denmark) (100 dB nHL) and SVV (5 repetitions). The ACS cVEMP, reflecting saccular function, showed normal latencies but a slightly abnormal amplitude asymmetry ratio (AR) of 40% (normal <33%). ACS cVEMP-AR was further reduced from week 1 until week 4 after therapy (Figures 2 and 3). BCV oVEMP latencies and AR were normal (<30%) before IGT administration, but AR was successively reduced from week 1 to 4 (Figure 4). SVV was normal (<2.5 degrees) before treatment and shifted to abnormal ranges to the affected side after weeks 2 and 3 (4.2 degrees and 3.8 degrees) but returned to normal values in week 4. The patient vestibular rehabilitation program started immediately at week 1, when IGT-induced vertigo occurred and change of hVOR and otolith function were proven in order to provide early central vestibular compensation. The compensational process went well; 1.5 years after IGT the patient still lacks any rotational vertigo attack.

## 3. Discussion

Disabling vertigo in MD can be treated with intratympanic gentamicin application, which is an evidence-based therapeutic option to reduce recurrent vertigo attacks. Reported success rates of vertigo control using several IGT protocols are >85% [1, 2]. Intratympanically applied, gentamicin is distributed by the round window to the perilymph in the vestibular organ and causes a damage of sensory epithelia, particularly type I hair cells, and nonsensory epithelia in order to reduce vestibular receptor function and to avoid endolymphatic hydrops [2, 3].

Impairment of semicircular canal (SCC) and otolith function (saccule and utricle) can now be recorded objectively and quantitatively by means of the vHIT, cVEMP, and oVEMP [4–7]. By means of recording eye/head velocity, the vHIT captures changes of high-frequency VOR and reflects therefore the VOR in its physiological way of ordinary head movements in the daily environment. Alterations of VOR are represented by a decrease in VOR gain and the occurrence of compensational refixation saccades (catch-up overt and/or catch-up covert saccades). Changes of hVOR can already be measured reliably with vHIT [6, 7].

Otolith function can be assessed by vestibular evoked myogenic potentials. Saccular function can be measured by ACS cVEMP, but BCV oVEMP reflects predominantly utricular function [8–10]. Changes in threshold levels, amplitude, or amplitude asymmetry as well as latency and latency differences are indicators of a disturbed saccular or utricular function. Since vHIT, cVEMP, and oVEMP represent receptor-specific, objective, and quantitative methods, they can be used to detect degraded vestibular receptor function as induced by the vestibulotoxic effect in IGT. Because in MD receptor function can already have deteriorated due to the disease itself, it is of clinical interest whether or not this degraded function can be further demolished by IGT to gain vertigo control. As demonstrated in our case, before IGT, vestibular receptor function was either normal (vHIT, BCV oVEMP) or already reduced (ACS cVEMP).

Data concerning evaluation of natural course in MD with vHIT is rare. A recent study revealed that the hVOR gain was normal in up to 45% of MD cases, whereas up to 55% exhibited a reduced hVOR gain (0.60 ± 0.20) with compensational refixation saccades. Distribution of refixation saccades was found to be 17% for covert, 33% for overt, and 50% for combined overt and covert saccades [11]. Furthermore, it has recently been shown that vHIT is able to separate acute MD attacks due to a gain enhancement from other peripheral vestibulopathies [12].

Until now, there is one single patient note concerning examination of hVOR using vHIT in IGT [13]. With search coil technique, Weber et al. have shown that high-frequency hVOR changes can occur after intravenous gentamicin [14]. Also with search coils, Carey et al. have demonstrated that IGT leads to a measurable and graded impairment of all SCC, but search coil technique is not suitable for daily clinical practice [15]. As demonstrated in our case, hVOR changes before and after IGT can reliably be recorded with the vHIT.

Compared to vHIT, a great number of studies exist on VEMP. The ACS cVEMP and oVEMP at 500 Hz is the standard diagnostic tool in MD [16–20]. At early stages of MD, saccular function may be already partially impaired, and as presented in our case, a mild impairment of otolith function can be identified using the cVEMPs at 500 Hz [17]. Furthermore, VEMP may enable separation of MD from other peripheral vestibulopathies [16, 19, 20].

In IGT at higher dosages it was confirmed by cVEMP that saccular function is impaired after therapy [21]. Helling et al. found in a low-dosage regimen that saccular function is impaired, but utricular function, recorded by SVV on eccentric rotation, appears to be maintained in up to 40% of cases [22]. Furthermore, especially the status of cVEMP at the second week after IGT seems to be a significant predictor of vertigo control [23].

The effect of IGT on the oVEMP has recently been evaluated in an animal study [24]. Since the vestibulotoxic effect in IGT is delayed and depends on several individual factors such as round window anatomy and middle ear mucosa thickness, several treatment protocols have been developed [1, 2, 25]. Changes of vestibular function in IGT have therefore to be considered under dynamic aspects, that is, *time* and *frequency* dependent.

Concerning the VOR, dynamic hVOR changes may be recorded by several methods such as thermal irrigation (low-frequency), rotational tests (low- and middle-frequency), and the clinical HIT and the vHIT (high-frequency). This frequency spectrum offers an overview concerning impaired dynamic hVOR working ranges in MD, it is one specific dynamic pattern of MD that the low-frequency hVOR is impaired whereas high-frequency tests still show normal results [26]. As in this case, before treatment hVOR showed a frequency-dynamic impairment of the hVOR with a pathologic side asymmetry of 36% in bithermal calorics (low-frequency vestibular testing lower than 0.1 Hz) but a normal vHIT (high-frequency vestibular testing with head impulses of 200°/s and up to 5 Hz). This result is analogical to the investigation of Black and Peterka using rotational tests, who recorded a decline of VOR function which was larger in lower-frequency stimuli compared to those in higher frequencies [27]. 

In contrast, Manzari et al. recently demonstrated short-term related dynamic changes of hVOR during acute MD attacks using the vHIT with an enhanced hVOR gain (>1). Therefore in acute vestibular syndromes such as MD, rotational vertigo can be distinguished from an attack induced by a vestibular neuritis, where hVOR gain is reduced [12, 20]. 

When hVOR function is not impaired in early MD stages, otolith function may be already altered [22, 23]. Therefore in IGT an insight into dynamic changes of otolith function is also important. Dynamic changes of otolith function may be objectified if VEMP are recorded using different stimulation frequencies (250–4000 Hz). Several works showed in patients with definite MD alterations in tuning responses by means of cVEMP and oVEMP with an amplitude shift up to 1000 Hz, which produces normally the largest amplitude using a 500 Hz stimulus [28]. With regard to dynamic changes during attacks it has been shown that utricular function can be enhanced, whereas saccular function was not affected [19]. However, Kuo et al. showed that during MD attacks saccular function was impaired in 67% [29].

In the presented case the patient is free of attacks 1.5 years after treatment, although it is well known that vertigo recurrences after IGT may occur. Low recurrence rates seem to depend on a low hVOR gain (0.4-0.5) after IGT [30]. Therefore, we emphasize the use of vHIT as an objective method to detect high-frequency changes of hVOR gain. With respect to dynamic control of otolith function it has recently been shown in single-shot, low-dose IGT regimen that cVEMPs were a significant predictor of posttreatment visual analog scale score, whereas a caloric test was not [23].

Since the outcome in vertigo control in different IGT protocols can hardly be predicted and several dynamic changes may occur before and after IGT, monitoring of dynamic changes of vestibular receptor function has to be recommended.

## 4. Conclusion

New tests such as vHIT, cVEMP and oVEMP provide an insight into vestibular receptor function. In combination with standard tests bithermal caloric irrigation and SVV, it is now possible to gain a dynamic view on the topological and functional status of vestibular receptors. Furthermore, with these complementary tests short-term and long-term dynamic changes of vestibular receptor function can be detected quantitatively and objectively. Therefore, SCC and otolith functions should be monitored in all therapeutic regimens, especially in IGT in order to gain permanent vertigo control.
